# ECL: an exhaustive search tool for the identification of cross-linked peptides using whole database

**DOI:** 10.1186/s12859-016-1073-y

**Published:** 2016-05-20

**Authors:** Fengchao Yu, Ning Li, Weichuan Yu

**Affiliations:** Division of Biomedical Engineering, The Hong Kong University of Science and Technology, Hong Kong, China; Division of Life Science, The Hong Kong University of Science and Technology, Hong Kong, China; Department of Electronic and Computer Engineering, The Hong Kong University of Science and Technology, Hong Kong, China

**Keywords:** Cross-linking, Peptide identification, Database searching

## Abstract

**Background:**

Chemical cross-linking combined with mass spectrometry (CX-MS) is a high-throughput approach to studying protein-protein interactions. The number of peptide-peptide combinations grows quadratically with respect to the number of proteins, resulting in a high computational complexity. Widely used methods including xQuest (Rinner et al., Nat Methods 5(4):315–8, 2008; Walzthoeni et al., Nat Methods 9(9):901–3, 2012), pLink (Yang et al., Nat Methods 9(9):904–6, 2012), ProteinProspector (Chu et al., Mol Cell Proteomics 9:25–31, 2010; Trnka et al., 13(2):420–34, 2014) and Kojak (Hoopmann et al., J Proteome Res 14(5):2190–198, 2015) avoid searching all peptide-peptide combinations by pre-selecting peptides with heuristic approaches. However, pre-selection procedures may cause missing findings. The most intuitive approach is searching all possible candidates. A tool that can exhaustively search a whole database without any heuristic pre-selection procedure is therefore desirable.

**Results:**

We have developed a cross-linked peptides identification tool named ECL. It can exhaustively search a whole database in a reasonable period of time without any heuristic pre-selection procedure. Tests showed that searching a database containing 5200 proteins took 7 h.

ECL identified more non-redundant cross-linked peptides than xQuest, pLink, and ProteinProspector. Experiments showed that about 30 *%* of these additional identified peptides were not pre-selected by Kojak. We used protein crystal structures from the protein data bank to check the intra-protein cross-linked peptides. Most of the distances between cross-linking sites were smaller than 30 Å.

**Conclusions:**

To the best of our knowledge, ECL is the first tool that can exhaustively search all candidates in cross-linked peptides identification. The experiments showed that ECL could identify more peptides than xQuest, pLink, and ProteinProspector. A further analysis indicated that some of the additional identified results were thanks to the exhaustive search.

**Electronic supplementary material:**

The online version of this article (doi:10.1186/s12859-016-1073-y) contains supplementary material, which is available to authorized users.

## Background

Chemical cross-linking combined with mass spectrometry (CX-MS) is becoming a powerful approach to studying protein-protein interactions. In the CX-MS protocol, proteins are linked before digestion. Digested products include cross-linked peptides and conventional linear peptides. In this paper, we refer to conventional linear peptides as peptides if there is no ambiguity. Cross-linked peptides are two peptides linked by a chemical compound. Two such peptides are referred to as chains, and the chemical compound is referred to as cross-linker. In the database searching based identification framework, the number of all possible peptide-peptide combinations grows quadratically with respect to the number of proteins, which results in a large search space.

Many tools have been developed to identify cross-linked peptides. An incomplete list includes ASAP [1], MS2Assign [2], MS-Bridge [3], CLPM [4], GPMAW [5], Virtual-MSLab [6], XDB [7], X!Link [8], Popitam [9], MS3D [10], CrossSearch [11], xComb [12], crux [13], Xlink-Identifier [14], pLink [27], Hekate [15], ProteinProspector [28, 29], Crossfinder [16], and Kojak [30]. The approach of most of these tools is to modify conventional peptide identification tools’ workflow and the corresponding score functions based on the property of cross-linked peptides. Because the search space is large, most of them pre-select high possibility candidates before scoring PSMs (peptide spectrum matches). In order to reduce the search space, cleavable cross-linkers [17–20] have been developed to avoid generating peptide-peptide combinations during database searching. Peptides linked by this kind of cross-linker can be broken into two peptides in dissociation. Thus, the cross-linked peptides identification problem is converted to the conventional peptide identification problem.

Due to the good chemical and biological properties of noncleavable amine-reactive cross-linkers (e.g. DSS (disuccinimidyl suberate) and BS3 (bis(sulfosuccinimidyl) suberate)), they have been widely used recently [21–24]. Tools including xQuest [25, 26], pLink [27], ProteinProspector [28, 29], and Kojak [30] were proposed to identify peptides linked by this kind of cross-linkers. They use preprocessing procedures to eliminate candidates with low possibilities before scoring. Given a spectrum, they compare it with the theoretical spectra from peptides to determine their chances of resulting in high scores heuristically. Peptides with low chances are eliminated. Eliminating some of the peptides before PSM scoring may result in false negatives. The most intuitive approach is searching all candidates exhaustively.

In this paper, we propose a new tool, named ECL (exhaustive cross-linked peptides identification), that can exhaustively search a whole database within a reasonable period of time. Experiments showed that more cross-linked peptides were identified thanks to exhaustive searching. For the purpose of visualization, we developed another tool, named ECLAnnotator, that converts ECL results into webpages. These webpages show annotated tandem mass spectra and matched/unmatched theoretical ions clearly.

## Implementation

ECL is designed to identify peptides linked by noncleavable amine-reactive cross linkers like DSS and BS3. In the current version, ECL only supports CID (collision-induced dissociation). Given a peptide-peptide combination, ECL *in silico* fragments it to b-ions and y-ions with different charges. These ions form a theoretical spectrum whose peaks’ intensities are the numbers of ions with the corresponding mass-to-charge ratios. The tandem mass spectra produced by a mass spectrometer are referred to as experimental spectra in this paper. ECL uses the normalized cross correlation coefficient to measure the similarity between a theoretical spectrum and an experimental spectrum:

1 $$  score = \frac{X^{T} Y}{||X|| ||Y||},  $$

where *X* is the theoretical spectrum, *Y* is the experimental spectrum, and *T* stands for vector transpose.

**Fig. 1:**
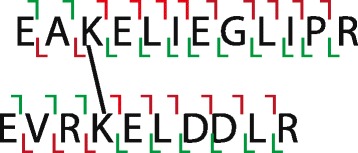
An illustration of cross-linked peptides’ dissociation pattern. Two chains’ lysines are linked. Green markers indicate linear ions, and red markers indicate cross-linking ions. A chain’s linear ions only contain that chain’s amino acids. A chain’s cross-linking ions contain that chain’s amino acids, a cross-linker, and another whole chain

Because the search space is large, we developed an efficient and low memory requirement algorithm to score PSMs. Concretely, Eq. (1) can be rewritten as:

2 $${} score = \frac{(X_{1} + X_{2})^{T} Y}{||X|| ||Y||} = \frac{{X_{1}^{T}} Y + {X_{2}^{T}} Y}{||X|| ||Y||} = \frac{{X_{1}^{T}} \tilde{Y} + {X_{2}^{T}} \tilde{Y}}{||X||},  $$

where *X*_1_ is the vector whose elements are contributed by the first chain, *X*_2_ is the vector whose elements are contributed by the second chain, *X*_1_+*X*_2_=*X*, and $\tilde {Y} = Y/||Y||$. ECL calculates $\tilde {Y}$ before scoring PSMs, which reduces the computational complexity largely. Both *X*_1_ and *X*_2_ have linear ions containing one chain’s amino acids and cross-linking ions containing both chains’ amino acids (Fig. 1). Given an experimental spectrum and a chain, ECL can obtain this chain’s ion masses as

3 $$ x_{i} = \left\{ \begin{array}{ll} p - c + l_{i}, & cross-linking\ ion \\ l_{i}, & linear\ ion \end{array} \right.,  $$

where *i* is the ion index starting from 0, *x*_*i*_ is *i*th ion’s mass, *p* is the experimental spectrum’s precursor mass, *c* is the chain’s mass, and *l*_*i*_ is the corresponding linear ion’s mass. Taking the first chain in Fig. 1 for example, 4th b-ion is a cross-linking ion containing “EAKE” and “EVRKELDDLR” linked by a cross-linker. Thus, its corresponding linear b-ion is “EAKE”. Clearly, *p*−*c* is equal to the summation of the other chain’s mass and the cross-linker’s mass. We don’t consider the difference between the experimental spectrum’s precursor mass and the theoretical spectrum’s precursor mass because the precursor mass tolerance is smaller than or equal to the tandem mass tolerance for almost all mass spectrometers. Given each ion’s mass, ECL calculates its corresponding mass-to-charge ratios with different charges. After getting all ions’ mass-to-charge ratios for one chain, ECL generates *X*_1_ or *X*_2_. Given an experimental spectrum, ${X_{1}^{T}} \tilde {Y}$ only needs to be calculated once for different *X*_2_, which reduces the computational complexity largely.

With the above optimization, ECL’s workflow is described as follows:

1. Indexing chains based on their masses.
2. Calculating ions’ masses for each chain.
3. Indexing experimental spectra based on their precursor masses.
4. Peak de-noising. Eliminating peaks whose intensities have the highest frequency.
5. Calculating $\tilde {Y} = Y/||Y||$ for each experimental spectrum.
6. Finding the largest precursor mass from all experimental spectra.
7. Looping over all chains whose masses are smaller than or equal to half of the largest precursor mass in ascending order:
  Finding all spectra whose precursor masses are larger than or equal to 2×*c*+*r*−*o*, where *r* is the cross-linker’s mass and *o* is the precursor mass tolerance.
  Calculating ions’ masses using Eq. (3), and using these masses to generate *X*_1_.
  Calculating ${X_{1}^{T}} \tilde {Y}$ for each corresponding spectrum.
  Finding all chains whose masses are within the range [*p*−*o*−*c*−*r, p*+*o*−*c*−*r*).
  Looping over all found chains:
    7.5.1 Calculating ions’ masses using Eq. (3), and using these masses to generate *X*_2_.
    7.5.2 Calculating ${X_{2}^{T}} \tilde {Y}$.
    7.5.3 Calculating the final score using Eq. (2).
    7.5.4 Saving each spectrum’s top score result as a PSM.
8. Estimating FDR (false discovery rate) for each PSM.
9. Converting FDR to *q*-value.

ECL estimates FDR as what xProphet [26] and pLink [27] do. Three kinds of PSMs are used:

1. Both chains are from the target database.
2. Both chains are from the decoy database.
3. One chain is from the target database and the other chain is from the decoy database.

FDR is estimated with

4 $$ FDR(s) = \frac{f(s) - d(s)}{t(s)},  $$

where *s* is a score, *t*(*s*) is the number of the first kind of PSMs whose scores are smaller than or equal to *s*, *d*(*s*) is the number of the second kind of PSMs whose scores are smaller than or equal to *s*, and *f*(*s*) is the number of the third kind of PSMs whose scores are smaller than or equal to *s*. Finally, FDR is converted to *q*-value [31]:

5 $$ q(t) = \min_{s \leq t} FDR(s),  $$

where *t* is a threshold.

## Results and discussion

### Computational complexity analysis

ECL is closely related to the work of Chen et al. [32] and Kojak [30]. Chen et al. [32] provided their algorithm’s computational complexity. Hoopmann et al. [30] provided Kojak’s source code without computational complexity analysis, so we analyzed its computational complexity based on the source code. In this section, we will analyze ECL’s computational complexity in detail.

#### Computational complexity analysis

Defining the following variables:

- *k*: number of proteins in a database.
- *n*: average number of peptides in a protein.
- *m*: average length of a chain.
- *h*: average number of peaks in an experimental spectrum.
- *s*: number of experimental spectra.
- *L*: number of precursor mass tolerance ranges. This approximately equals the precursor mass range divided by the precursor mass tolerance.

The time complexity of the algorithm proposed by Chen et al. [32] is

6 $$  O(skn^{2} \log (kn) + sk^{2}n^{2} \log (kn) / L + s k^{2} n^{2}(m + h) / L).  $$

For the first and second terms, the authors only considered one experimental spectrum. We multiply the terms by *s* because there are *s* experimental spectra. We also use *k*^2^*n*^2^/*L* to replace *p* in the original paper. For the third term, the authors only considered one PSM. We multiply the term by *sk*^2^*n*^2^/*L* because there are *k*^2^*n*^2^/*L* peptide-peptide combinations for each experimental spectrum and there are *s* experimental spectra. The time complexity of Kojak is

7 $$  O(kn \log(s) + kns (m + h + 1) + s t^{2}).  $$

Please refer to the Additional file 1 for details.

For ECL, the computational complexity is dominated by step 7 in the workflow. The complexity of step 7.1 is *O*(log(*s*)). Steps 7.2 and 7.5.1 have the same time complexity, *O*(*m*). ECL stores theoretical and experimental spectra in sparse matrixes. We developed an algorithm to match peaks between a theoretical spectrum and an experimental spectrum with *O*(*m*+*h*) complexity (Algorithm 1). Thus, both steps 7.3 and 7.5.2 have the time complexity, *O*(*m*+*h*). Moreover, for an experimental spectrum and a pair of chains, steps 7.2 and 7.3 only need to be executed once because ECL checks each chain whose mass is smaller than or equal to half of the largest precursor mass in ascending order. Steps 7.3 and 7.5.2 also only need to be executed once for the same reason. The time complexity of step 7.4 is *O*(log(*kn*)). The time complexity of steps 7.5.3 and 7.5.4 is *O*(*kns*/*L*). Thus, the time complexity of step 7 is

8 $$ O(kn(\log(s) + m + s(m + h) + \log(kn) + kns / L)).  $$

There are seven variables in the time complexity equations. Five of them can be fixed based on biological prior knowledge:

- *n*≈100.
- *m*≈20.
- *h*≈10^2^.
- *s*≈10^4^.
- *L*≈10^5^.

**Fig. 2:**
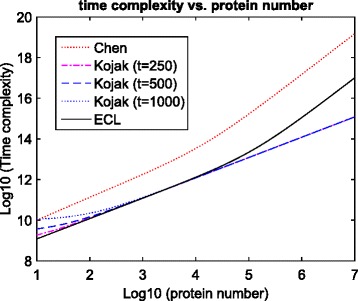
Computational complexity against different numbers of proteins. Three *t* values were used to plot Kojak’s computational complexity curves. Chen et al. [32] has the highest time complexity. When the number of proteins is small, ECL has smaller time complexity compared to Kojak. When the number of proteins is large, ECL has higher complexity than Kojak

We plotted curves of Eqs. (6), (7), and (8) against different numbers of proteins (Fig. 2). Since Kojak selects *t* peptides for each spectrum, we plotted three curves corresponding to three different *t* values. We can see that Chen et al. [32] has the highest time complexity. When the number of proteins is small, ECL has smaller time complexity compared to Kojak (leftmost of Fig. 2). This is because ECL doesn’t need to select peptides beforehand. When the number of protein is large, ECL has higher complexity than Kojak (rightmost of Fig. 2). This is because the number of peptide-peptide combinations searched by ECL grows quadratically as the increase of protein number (Eq. (8)). This is an unavoidable cost of exhaustive searching. On the other hand, the number of peptide-peptide combinations searched by Kojak is almost constant, and the total time complexity increases linearly (Eq. (7)).

Even though ECL’s time complexity is large, it can still handle a large database. Given a data set containing thousands of tandem mass spectra, ECL only needs 7 h to search a database containing 5200 proteins.

#### Space complexity

- The space complexity of Chen et al. [32] is
  9 $$  O(kn + k^{2} n^{2}/L + knm + h).  $$
  For the second term, we use *k*^2^*n*^2^/*L* to replace *p* in the original paper. For the third term, the authors only considered one peptide-peptide combination for each experimental spectrum. We multiply the term by *kn* considering that there are *kn* peptides for each experimental spectrum.
- There are two steps in Kojak. The space complexity of the first step is *O*(*m*+*sh*), and the space complexity of the second step is *O*(*tm*+*h*). Thus, the total space complexity is
  10 $$ O(m + sh + tm + h).  $$
- The space complexity of ECL is
  11 $$  O(knm + sh).  $$

Clearly, Chen et al. [32] has the highest space complexity, and Kojak has the lowest space complexity. Although ECL’s space complexity is higher than that of Kojak, from our experience, a personal computer with 32G memory is sufficient in most cases.

### Experiments

In this paper, we will present two sets of experiments. The first one used a data set from the cross-linking of two synthetic peptides. The second one used four data sets from the 26S proteasome sample [33] provided by xQuest [25, 26]. Since our study did not involve any humans, animals or clinical data, we do not have ethics or consent issues.

#### An experiment with synthetic peptides

This experiment used two synthetic peptides produced by GL Biochem (Shanghai) Ltd. The sequences were “EVRKELDDLR” and “EAKELIEGLPR”. N-terminals were protected by Fmoc. We used 1 *μ*L peptides and 0.5 *μ*L DSS. Their concentrations were 1 and 0.5 mM, respectively. We dissolved the peptides and DSS in DMSO (dimethyl sulfoxide) to a final concentration of 50 mM. The reaction was carried out at room temperature, and the reaction time was 2 h. After quenching, we added 12.5 *μ*L piperidine to the above solution to remove the Fmoc protection. The reaction lasted for another 2 h. Finally, we freeze-dried the sample to obtain the cross-linked peptides.

LC-MS (liquid chromatography-mass spectrometry) analysis was carried out on a Thermo LTQ Orbitrap XL mass spectrometer (Thermo Fisher Scientific Inc.) with a NanoLC system. The sample was loaded onto a trapping column (PepMap C18; 2 cm × 100 *μ*m × 5 *μ*m, 100 Å) using a flow rate of 4 *μ*L/min of solvent A. The loading lasted for 10 min. Cross-linked peptides were separated at a flow rate of 200 L/min on a 75 *μ*m × 50 cm C18 column (Acclaim PepMap RSLC C18, 75 *μ*m × 50 cm × 3 *μ*m, 100 Å). The following gradient was used: 0–8 min 2 % B, 8–12 min 2–10 % B, 12–180 min 10–50 % B, 180–200 min 50–98 % B, 200–215 min 98 % B, and 215–240 min 98 – 2 % B, where B was the ratio of acetonitrile to formic acid. B equaled 100:0.1 in this experiment. The mass spectrometer selected up to five precursors to perform CID. The intensity threshold of triggering fragmentation was 150 counts. Only those whose precursor charges were larger than or equal to 2 were considered. CID was performed for 30 ms using 35 % normalized collision energy and a 0.25 activation value. Dynamic exclusion was used with the following parameters: 1 repeat count, 60 s exclusion duration, 500 list size, and 10 ppm mass window. The ion target value was 1,000,000 (or 500 ms fill time) for full scans, and 1,000,000 (or 200 ms fill time) for a tandem mass scan. Fragmented ions were detected in a linear ion trap.

During the search, the precursor mass tolerance was 10ppm, and the tandem mass tolerance was 0.5Th. Up to 2 missed cleavages were allowed. The database contained 100 randomly selected proteins and two synthetic peptides. The decoy database was generated by reversing peptides, with lysine and arginine fixed. Because there was only one linkable site in each synthetic peptide, all cross-linked peptides formed by synthetic peptides were treated as inter-protein cross-linked peptides. The *q*-value cut-off threshold was 0.05.

The search was carried out on a personal computer with an Intel Core i5-4570 CPU (central processing unit) and 32 GB memory. ECL needed about 100 s to finish the task. Since we knew the ground truth, we could calculate the false discovery proportion. 4 out of 149 PSMs were incorrect. The corresponding false discovery proportion was 0.03. This experiment indicated that ECL could provide trustable results. Details can be found in the Additional file 2.

#### Experiments with 26S proteasome data

Four data sets from the 26S proteasome sample [25, 26, 33] were used. We first searched four data sets against a database released along with the data sets. It contained 34 proteins. The latest versions of xQuest, pLink, ProteinProspector, Kojak, and ECL were used: xQuest 2.1.1, pLink 1.23, ProteinProspector 5.14.4, Kojak 1.4.2, and ECL 20160117. The precursor mass tolerance was 10 ppm, and the tandem mass tolerance was 0.2Da. Other parameters were the same as those in the previous experiment. All the parameter files used by these tools were included in the Additional file 3. We used xProphet [26] to estimate the *q*-value for xQuest’s results by setting “qtransform” to 1 in the “xproph.def” file. Because ProteinProspector did not provide the *q*-value in its results, we estimated it as what Trnka et al. [29] did. We used Percolator to estimate the *q*-value for Kojak’s results as what Kojak required. Intra-protein cross-linked peptides and inter-protein cross-linked peptides were analyzed separately. For a fair comparison, these tools’ *q*-value thresholds were 0.05.

Table 1 shows the numbers of non-redundant cross-linked peptides identified by xQuest, pLink, ProteinProspector, Kojak, and ECL, respectively. Corresponding Venn diagrams can be found in the Additional file 1. ECL identified more cross-linked peptides than xQuest, pLink, and ProteinProspector. We used protein crystal structures from the protein data bank (PDB) to measure the distances between linking-sites in intra-protein cross-linked peptides. Only 3 proteins had structural information. Their UniProt accessions were O94444, P06732, and P50524, respectively. The corresponding PDB ID were 2X5N, 1I0E, and 4B0Z, respectively. There were 65 PSMs to these proteins. 60 of them had a distance smaller than 30 Å, which meant that they were within the distance tolerance. Details can be found in the Additional file 4. We also used ECLAnnotator to generate annotated tandem mass spectra for ECL’s results. They can be found at http://bioinformatics.ust.hk/ecl.html. Then, we analyzed matched and unmatched peaks. Please refer to the Additional file 2 for details.

**Table 1:** Numbers of non-redundant cross-linked peptides identified by xQuest, pLink, ProteinProspector, Kojak, and ECL, respectively. The database contains 34 proteins

Data set	xQuest	pLink	ProteinProspector	Kojak	ECL
1	70 (56)	5 (4)	104 (69)	102 (71)	97
2	73 (41)	28 (17)	99 (45)	120 (56)	58
3	90 (62)	28 (10)	96 (64)	139 (90)	127
4	61 (47)	20 (14)	94 (68)	110 (83)	135

Values in the brackets are the numbers of overlapping cross-linked peptides identified by both ECL and the corresponding method

In order to find out if the additionally identified cross-linked peptides were due to exhaustive search, we let Kojak output top 9999 pre-selected peptides for each cross-linked peptide’s highest score spectrum. (The default number of pre-selected peptides is 250. To our knowledge, other tools can not output their pre-selected peptides). Then, we compared the cross-linked peptides identified by ECL with those pre-selected peptides in the corresponding spectra. We consider one additionally identified cross-linked peptides pair is due to exhaustive search if all of the following criteria are satisfied (We thank the anonymous reviewer for suggesting these criteria):

1. The precursor masses in Kojak and ECL are within the same tolerance range.
2. If both of two peptide chains are in the pre-selection list and at least one is over 250, Kojak and ECL identify the same pair of peptide chains.
3. At least one peptide chain isn’t in the pre-selection list.

Table 2 shows the summarized results. About 30 *%* of these peptides aren’t within top 250 of Kojak’s pre-selected peptides, which means that the pre-selection procedure is one of the causes of missing findings. Each spectrum’s pre-selected peptides and detailed comparison results can be found in the Additional file 5.

**Table 2:** A table showing if Kojak searched those missing identified peptides

Data set	Number of peptides from the cross-linked peptides identified by ECL, but not by Kojak	Number of peptides that don’t belong to Kojak’s pre-selected peptides	Ratio
1	25	2	0.08
2	2	1	0.50
3	37	12	0.32
4	52	21	0.40
Total	116	36	0.31

The second column contains the total numbers of peptides from the cross-linked peptides identified by ECL, but not by Kojak. The third column contains the numbers of peptides that don’t belong to Kojak’s pre-selected peptides. The forth column contains the ratios between the number in the third column and the number in the second column

Table 3 shows the corresponding running time of xQuest, pLink, Kojak, and ECL, respectively. ProteinProspector spent 1254 seconds on average analyzing one data set. It was run on the authors’ web server so we didn’t compare it with the other four tools. Since Kojak supports multi-thread computing, we ran it with 4 threads. xQuest, pLink, and ECL don’t support multi-thread computing.

**Table 3:** Running time of xQuest, pLink, Kojak, and ECL, respectively. The unit is second

Data set	xQuest	pLink	Kojak (4 threads)	ECL
1	6349	851	46	51
2	6741	878	48	57
3	20419	876	49	60
4	21757	700	47	60

Finally, we tested if ECL could search a large database within a reasonable period of time. We searched the same data sets against the whole proteome of Schizosaccharomyces pombe species. There were 5200 proteins. We set the allowed maximum missed cleavage to 1. The rest of the parameters were the same as those in the last experiment. xQuest ran for a few days, but it still couldn’t finish the searching. pLink could not handle such a large database. ProteinProspector spent 1.7 h on average analyzing one data set on the authors’ web server. Kojak spent 0.25 h on average analyzing one data set. ECL spent 7 h on average analyzing one data set.

There were 4×10^10^ peptide-peptide combinations including decoy peptides. The precursor mass tolerance was 10 ppm. Thus, there were about 4×10^5^ peptide-peptide combinations for each spectrum. Kojak selected top 250 peptides to generate peptide-peptide combinations for each spectrum, which covered about 8 *%* of the whole search space. ProteinProspector used a similar pre-selection procedure to select top 1000 peptides. Thus, the number of peptide-peptide combinations searched by ProteinProspector and Kojak was almost constant with the increase of the database size. However, the number of peptide-peptide combinations searched by ECL increased quadratically. That’s why ECL was slower than ProteinProspector and Kojak.

ProteinProspector, Kojak, and ECL identified fewer cross-linked peptides compared with the previous experiment (Table 4). It is a known issue [34, 35] that larger databases lead to fewer results. The discussion of this issue is beyond the scope of this paper. ECL identified more non-redundant peptides than ProteinProspector and Kojak. Please note that there is no intra-protein cross-linked peptides identified by Kojak because Percolator output errors in estimating *q*-value for Kojak. The errors said: “the input data has too good separation between target and decoy PSMs”. It is a common error when there are only a few target or decoy PSMs. Please refer to Percolator’s document for more detail.

**Table 4:** Numbers of non-redundant cross-linked peptides identified by ProteinProspector, Kojak, and ECL, respectively. The database contains 5200 proteins

Data set	ProteinProspector	Kojak	ECL
1	20 (15)	5 (0)	36
2	32 (16)	6 (0)	39
3	24 (12)	4 (0)	39
4	23 (17)	2 (0)	57

Values in the brackets are the numbers of overlapping cross-linked peptides identified by both ECL and the corresponding method. There is no result for intra-protein cross-linked peptides reported by Kojak because Percolator outputs errors in estimating *q*-value

## Conclusions

High computational complexity is a major obstacle in exhaustively carrying out large-scale cross-linked peptides identification. To the best of our knowledge, ECL is the first tool that successfully addresses the computational complexity issue without any heuristic pre-selection procedure. Given thousands of tandem mass spectra and a database containing thousands of proteins, it can finish the task in a few hours. The experiments showed that ECL could identify more peptides than xQuest, pLink, and ProteinProspector. A further analysis on public data sets showed that exhaustive search helped identify more cross-linked peptides than existing methods.

## Availability and requirements

**Project name:** ECL**Project home pase:**http://bioinformatics.ust.hk/ecl.html**Operating systems:** Windows, Linux, OS X**Programming language:** Java, Python**Other requirements:** Java 1.7 or higher, Python 2.7**License:** Apache License 2

## Additional files

Additional file1 A supplementary document contains ECL user instruction, computational complexity analysis of Kojak, spectra analysis of the 26S Proteasome results, and venn diagrams of 26S Proteasome results. (PDF 434 kb)

Additional file 2 Detailed results of synthetic peptides and 26S Proteasome samples. (ZIP 4432 kb)

Additional file 3 Parameter files used by xQuest, ProteinProspector, Kojak, and ECL, respectively. (ZIP 17 kb)

Additional file 4 Distances of intra protein identified by ECL. (XLSX 15 kb)

Additional file 5 Kojak’s pre-selection list of PSMs only identified by ECL. (ZIP 5147 kb)

## Abbreviations

BS3: Bis(sulfosuccinimidyl) suberate. CID: collision-induced dissociation
CPU: central processing unit
CX-MS: chemical cross-linking combined with mass spectrometry
DMSO: dimethyl sulfoxide
DSS: disuccinimidyl suberate
ECL: exhaustive cross-linked peptides identification tool
ETD: electron-transfer dissociation
FDR: false discovery rate
PDB: protein data bank
PSM: peptide spectrum match
