# Supplementary material for: ECL: an exhaustive search tool for the identification of cross-linked peptides using whole database
Source: BMC Bioinformatics. 2016 May 20;17:217. doi: 10.1186/s12859-016-1073-y (PMC4874008; doi:10.1186/s12859-016-1073-y)
Supplement: Additional file1 — A supplementary document contains ECL user instruction, computational complexity analysis of Kojak, spectra analysis of the 26S Proteasome results, and venn diagrams of 26S Proteasome results. (PDF 434 kb) [file 12859_2016_1073_MOESM1_ESM.pdf]

ECL: An Exhaustive Search Tool for the Identification of Cross-Linked Peptides Using Whole Database

Fengchao Yu, Ning Li, and Weichuan Yu

1 How to Use ECL

The running command is

```
java -Xmx32g -jar ECL.jar parameter.def data.mzXML
```

where “parameter.def” is a parameter file containing all parameters, “data.mzXML” is a mass spectra file. There are three outputs:

1. “data.mzXML.intra.csv”,
2. “data.mzXML.inter.csv”,
3. “ECL.log”,

The first file contains PSMs (peptide spectrum matches) belonging to intra protein cross-linked peptides, the second file contains PSMs belonging to inter protein cross-linked peptides, and the last file is a log. The first two files can be opened with Excel. Columns in the first two files are arranged as follows:

1. Scan number.
2. The spectrum’s precursor mass-to-charge ratio.
3. The spectrum’s precursor charge.
4. Score.
5. The ratio between the second ranked score and the top ranked score.
6. The derivation between the spectrum’s precursor mass and theoretical precursor mass.
7. Sequence of the first chain.
8. Cross-linking site of the first chain.
9. Modifications in the first chain.
10. The corresponding protein of the first chain.
11. The protein annotation of the first chain.
12. Sequence of the second chain.
13. Cross-linking site of the second chain.
14. Modification in the second chain.
15. The corresponding protein of the second chain.
16. The protein annotation of the second chain.
17. q-value.

2 How To Use ECLAnnotator

ECLAnnotator is a tool converting results into webpages. The running command is

```
python ECLAnnotator.py parameter.def result.csv data.mzXML
```

where "parameter.def" is a parameter file used by ECL, "result.csv" is the result of ECL, "data.mzXML" is a mass spectra file. The output contains a folder named "figures" and a html file named "result.csv.html". Opening the html file with a browser, we can see a list of results (Figure S1). The final column contains links pointing to annotated spectra (Figure S2).

ECL Result

aleitner_M1012_005.mzXML

inter-protein

<table>
<tr><th>scan_num</th><th>charge</th><th>score</th><th>q_value</th><th>peptide_1</th><th>site_1</th><th>peptide_2</th><th>site_2</th><th>protein_1</th><th>protein_2</th><th>ions</th><th>spectrum</th></tr>
<tr><td>2597</td><td>4</td><td>0.30613723398</td><td>0.0</td><td>KVPDK</td><td>1</td><td>KLEGTIDYQK</td><td>1</td><td>sp|O94517|PSA6_SCHPO</td><td>sp|O74445|PRS10_SCHPO</td><td>37/575</td><td>Spectrum</td></tr>
<tr><td>4596</td><td>4</td><td>0.282370900985</td><td>0.0</td><td>YKVNLYK</td><td>2</td><td>KIYNEYPPTK</td><td>1</td><td>sp|Q9Y7T8|PSB3_SCHPO</td><td>sp|O94579|PSA2_SCHPO</td><td>33/566</td><td>Spectrum</td></tr>
<tr><td>4676</td><td>3</td><td>0.263885055773</td><td>0.0</td><td>YKVNLYK</td><td>2</td><td>KIYNEYPPTK</td><td>1</td><td>sp|Q9Y7T8|PSB3_SCHPO</td><td>sp|O94579|PSA2_SCHPO</td><td>26/742</td><td>Spectrum</td></tr>
<tr><td>4769</td><td>4</td><td>0.263700269868</td><td>0.0</td><td>YKVNLYK</td><td>2</td><td>KIYNEYPPTK</td><td>1</td><td>sp|Q9Y7T8|PSB3_SCHPO</td><td>sp|O94579|PSA2_SCHPO</td><td>33/663</td><td>Spectrum</td></tr>
<tr><td>6405</td><td>4</td><td>0.263322762376</td><td>0.0</td><td>KVAQTTYK</td><td>1</td><td>SEAAEFEYKNGYPMPCDVLAK</td><td>9</td><td>sp|O94579|PSA2_SCHPO</td><td>sp|O94517|PSA6_SCHPO</td><td>42/770</td><td>Spectrum</td></tr>
<tr><td>4855</td><td>4</td><td>0.256439340644</td><td>0.0</td><td>YKVNLYK</td><td>2</td><td>KIYNEYPPTK</td><td>1</td><td>sp|Q9Y7T8|PSB3_SCHPO</td><td>sp|O94579|PSA2_SCHPO</td><td>36/745</td><td>Spectrum</td></tr>
<tr><td>4590</td><td>3</td><td>0.251365843306</td><td>0.0</td><td>YKVNLYK</td><td>2</td><td>KIYNEYPPTK</td><td>1</td><td>sp|Q9Y7T8|PSB3_SCHPO</td><td>sp|O94579|PSA2_SCHPO</td><td>25/773</td><td>Spectrum</td></tr>
<tr><td>9478</td><td>3</td><td>0.245287787943</td><td>0.0</td><td>SKTLQYFA</td><td>2</td><td>KVEFGLPDLEGR</td><td>1</td><td>sp|O14126|PRS6A_SCHPO</td><td>sp|O42931|PRS7_SCHPO</td><td>31/840</td><td>Spectrum</td></tr>
<tr><td>3955</td><td>3</td><td>0.240160259165</td><td>0.0</td><td>VVKGNQK</td><td>3</td><td>KILFENPDEATK</td><td>1</td><td>sp|O42931|PRS7_SCHPO</td><td>sp|P36612|PRS4_SCHPO</td><td>31/735</td><td>Spectrum</td></tr>
<tr><td>4941</td><td>4</td><td>0.239671589852</td><td>0.0</td><td>YKVNLYK</td><td>2</td><td>KIYNEYPPTK</td><td>1</td><td>sp|Q9Y7T8|PSB3_SCHPO</td><td>sp|O94579|PSA2_SCHPO</td><td>39/805</td><td>Spectrum</td></tr>
<tr><td>5231</td><td>3</td><td>0.231051465888</td><td>0.0</td><td>TFLEKR</td><td>5</td><td>VTSKLLEQEESAEK</td><td>4</td><td>sp|O94579|PSA2_SCHPO</td><td>sp|Q09682|PSA4_SCHPO</td><td>28/892</td><td>Spectrum</td></tr>
<tr><td>4935</td><td>3</td><td>0.230303872159</td><td>0.0</td><td>YKVNLYK</td><td>2</td><td>KIYNEYPPTK</td><td>1</td><td>sp|Q9Y7T8|PSB3_SCHPO</td><td>sp|O94579|PSA2_SCHPO</td><td>25/1016</td><td>Spectrum</td></tr>
<tr><td>4508</td><td>4</td><td>0.228790155533</td><td>0.0</td><td>YKVNLYK</td><td>2</td><td>KIYNEYPPTK</td><td>1</td><td>sp|Q9Y7T8|PSB3_SCHPO</td><td>sp|O94579|PSA2_SCHPO</td><td>31/705</td><td>Spectrum</td></tr>
<tr><td>6409</td><td>3</td><td>0.225788990126</td><td>0.0</td><td>KVAQTTYK</td><td>1</td><td>SEAAEFEYKNGYPMPCDVLAK</td><td>9</td><td>sp|O94579|PSA2_SCHPO</td><td>sp|O94517|PSA6_SCHPO</td><td>33/951</td><td>Spectrum</td></tr>
<tr><td>3262</td><td>4</td><td>0.217256142324</td><td>0.0</td><td>KLGVKPPK</td><td>1</td><td>IHLEKVSK</td><td>5</td><td>sp|O14126|PRS6A_SCHPO</td><td>sp|O74445|PRS10_SCHPO</td><td>33/685</td><td>Spectrum</td></tr>
<tr><td>5837</td><td>4</td><td>0.213558399784</td><td>0.0</td><td>KIEFPSYR</td><td>1</td><td>VLNKGDSGEMSLQK</td><td>4</td><td>sp|O74894|PRS6B_SCHPO</td><td>sp|P41836|PRS8_SCHPO</td><td>43/898</td><td>Spectrum</td></tr>
<tr><td>4763</td><td>3</td><td>0.20857275987</td><td>0.0</td><td>YKVNLYK</td><td>2</td><td>KIYNEYPPTK</td><td>1</td><td>sp|Q9Y7T8|PSB3_SCHPO</td><td>sp|O94579|PSA2_SCHPO</td><td>26/815</td><td>Spectrum</td></tr>
<tr><td>7881</td><td>4</td><td>0.208270475633</td><td>0.0</td><td>KQLATSLR</td><td>1</td><td>TFANLVSSTLYEKR</td><td>13</td><td>sp|Q09720|PSB2_SCHPO</td><td>sp|Q9Y7T8|PSB3_SCHPO</td><td>29/794</td><td>Spectrum</td></tr>
<tr><td>5534</td><td>4</td><td>0.202482505428</td><td>0.0</td><td>IQYVGKQDPK</td><td>6</td><td>ELLKPSASVALQR</td><td>4</td><td>sp|P41878|RPN11_SCHPO</td><td>sp|O74894|PRS6B_SCHPO</td><td>33/929</td><td>Spectrum</td></tr>
<tr><td>6215</td><td>4</td><td>0.202268275777</td><td>0.0</td><td>LMSKVAEK</td><td>4</td><td>LLEKLEIK</td><td>4</td><td>sp|O14250|PSA1_SCHPO</td><td>sp|Q9USQ9|PSB4_SCHPO</td><td>24/720</td><td>Spectrum</td></tr>
<tr><td>5540</td><td>3</td><td>0.200872658432</td><td>0.0</td><td>IQYVGKQDPK</td><td>6</td><td>ELLKPSASVALQR</td><td>4</td><td>sp|P41878|RPN11_SCHPO</td><td>sp|O74894|PRS6B_SCHPO</td><td>24/912</td><td>Spectrum</td></tr>
<tr><td>4378</td><td>3</td><td>0.200203169332</td><td>0.0</td><td>KVPDK</td><td>1</td><td>KLEGTIDYQKL</td><td>10</td><td>sp|O94517|PSA6_SCHPO</td><td>sp|O74445|PRS10_SCHPO</td><td>27/918</td><td>Spectrum</td></tr>
<tr><td>4848</td><td>3</td><td>0.199229989754</td><td>0.0</td><td>YKVNLYK</td><td>2</td><td>KIYNEYPPTK</td><td>1</td><td>sp|Q9Y7T8|PSB3_SCHPO</td><td>sp|O94579|PSA2_SCHPO</td><td>27/886</td><td>Spectrum</td></tr>
<tr><td>1886</td><td>3</td><td>0.196910918008</td><td>0.04</td><td>AQEEVKR</td><td>6</td><td>KHLDAEVQK</td><td>1</td><td>sp|O74894|PRS6B_SCHPO</td><td>sp|P41878|RPN11_SCHPO</td><td>25/804</td><td>Spectrum</td></tr>
<tr><td>3460</td><td>4</td><td>0.195982116701</td><td>0.04</td><td>LGVKPPK</td><td>4</td><td>IHLEKVSK</td><td>5</td><td>sp|O14126|PRS6A_SCHPO</td><td>sp|O74445|PRS10_SCHPO</td><td>22/608</td><td>Spectrum</td></tr>
</table>

Figure S1: A screenshot of the main webpage.

3 Computational Complexity Analysis of Kojak

There are two parts in Kojak's algorithm. The first one is a procedure selecting top 250 peptides for each spectrum. Given a peptide, Kojak finds all spectra whose precursor masses are within a wide range. The time complexity for all peptide is $O(kn \log(s))$. Then, Kojak calculates $XCorr$ for each pair of a peptide and an experimental spectrum:

$$XCorr(X, Y) = X_0 \cdot Y_0 - \left(\sum_{\tau=-75}^{\tau=75} X_0 \cdot Y_\tau \right) / 151, \tag{1}$$

where X is the theoretical spectrum of the peptide, Y is the experimental spectrum, X_0 is the theoretical spectrum with shift 0, and Y_τ is the experimental spectrum with shift τ. Kojak uses a fast algorithm proposed by Eng *et al.* [1, 2] to calculate Equation (1). Its time complexity for one pair is $O(m + h)$, and there are $O(kns)$ pairs. After obtaining a $XCorr$, Kojak inserts it into a sorted array containing all $XCorr$ corresponding to the experimental spectrum. The time complexity for each experimental spectrum is $O(kn)$, and there are $O(s)$ experimental spectra. Thus, the time complexity of the first part is

$$O(kn \log(s) + kns(m + h) + kns). \tag{2}$$

The second part calculates a final score for each pair of an experimental spectrum and a cross-linking theoretical spectrum. Kojak uses the result of the first part to calculate the final score. The time complexity is

$$O(st^2). \tag{3}$$

Summing Equation (2) and Equation (3) together, we have the total time complexity

$$O(kn\log(s) + kns(m + h + 1) + st^2). \tag{4}$$

4 Annotated Spectra Analysis of the 26S Proteasome Results

Kojak and ECL identified hundreds of non-redundant cross-linked peptides with four 26S proteasome data sets. We picked the highest score spectrum for each cross-linked peptides, and summarized the ratios of matched peaks' intensities versus all peaks' intensities. Then, we compared the ratios from the spectra identified both by ECL and Kojak with the ratios from the spectra identified by ECL only. Their histograms are shown in Figure S3. We can see that these two sets of ratios have a similar distribution, which means that those additional identified cross-linked peptides have the same matching quality as that of overlapped cross-linked peptides.

5 Venn Diagrams of the 26S Proteasome Results

We plotted Venn diagrams for four data sets with non-redundant cross-linked peptides (Figure S4).

References

[1] J. K. Eng, B. Fischer, J. Grossmann, and M. J. MacCoss, "A fast SEQUEST cross correlation algorithm," *Journal of Proteome Research*, vol. 7, no. 10, pp. 4598–4602, 2008.

[2] J. K. Eng, T. A. Jahan, and M. R. Hoopmann, "Comet: An open-source MS/MS sequence database search tool," *Proteomics*, vol. 13, no. 1, pp. 22–24, 2013.

aleitner_M1012_005.mzXML.4596.4

Annotated spectrum

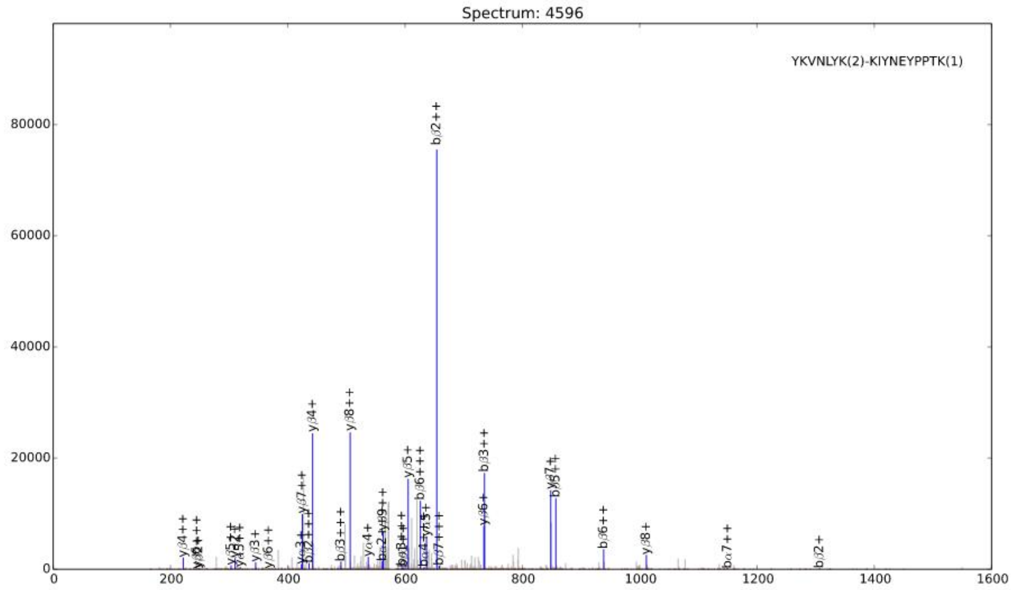

Theoretical ions table

Alpha chain

	Y	K	V	N	L	Y	K
b_charge_1	164.07	1681.88	1780.95	1894.99	2008.08	2171.14	2299.24
y_charge_1	147.11	310.18	423.26	537.30	636.37	2154.18	2317.25
b_charge_2	82.54	841.45	890.98	948.00	1004.54	1086.07	1150.12
y_charge_2	74.06	155.59	212.13	269.16	318.69	1077.60	1159.13
b_charge_3	55.36	561.30	594.32	632.34	670.03	724.39	767.08
y_charge_3	49.71	104.06	141.76	179.77	212.80	718.73	773.09

Beta chain

	K	I	Y	N	E	Y	P	P	T	K
b_charge_1	1193.69	1306.78	1469.84	1583.88	1712.93	1875.99	1973.04	2070.09	2171.14	2299.24
y_charge_1	147.11	248.16	345.21	442.27	605.33	734.37	848.41	1011.48	1124.56	2317.25
b_charge_2	597.35	653.89	735.42	792.45	856.97	938.50	987.02	1035.55	1086.07	1150.12
y_charge_2	74.06	124.58	173.11	221.64	303.17	367.69	424.71	506.24	562.78	1159.13
b_charge_3	398.57	436.26	490.62	528.63	571.65	626.00	658.35	690.70	724.39	767.08
y_charge_3	49.71	83.39	115.74	148.09	202.45	245.46	283.48	337.83	375.53	773.09

Figure S2: A screenshot of a annotated spectrum and theoretical ions.

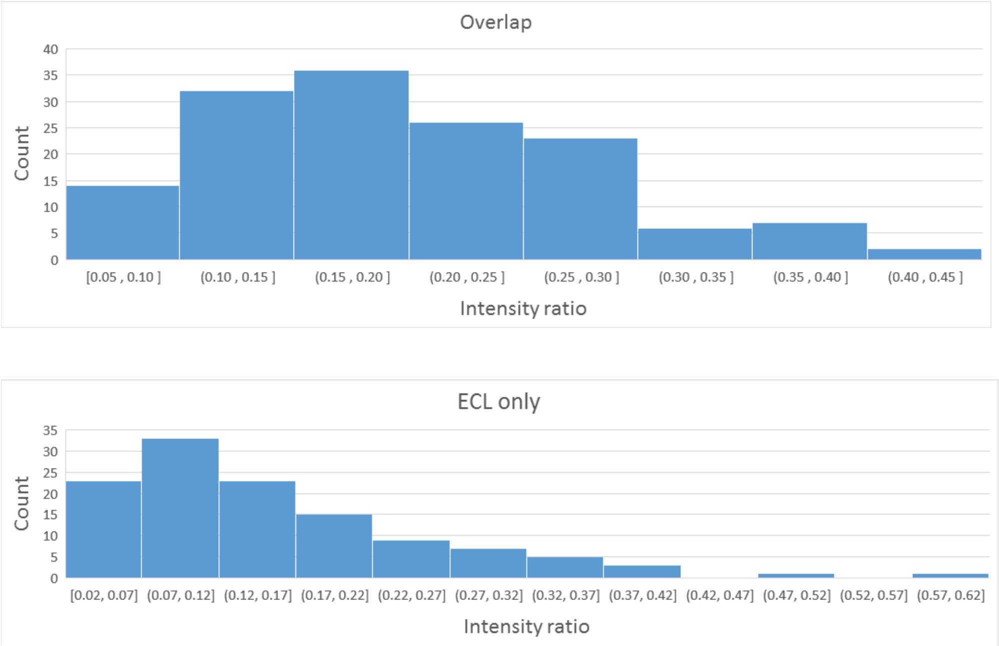

Figure S3: Histograms of the ratios of matched peaks' intensities versus all peaks' intensities. The ratios of the first histogram are from the spectra identified by both Kojak and ECL. The ratios of the second histogram are from the spectra identified by ECL only.

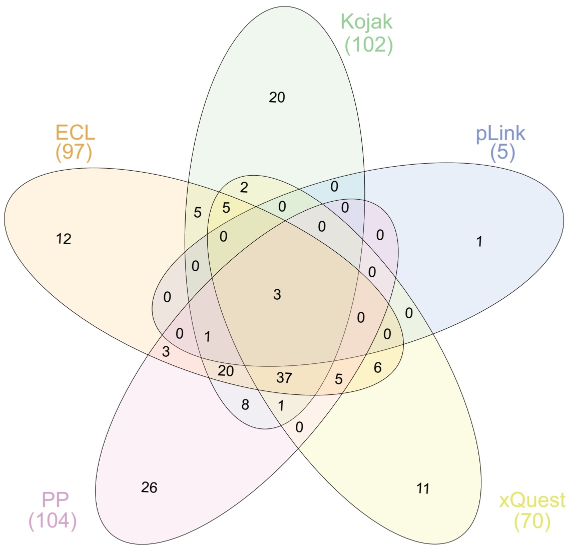

(a) Data set 1

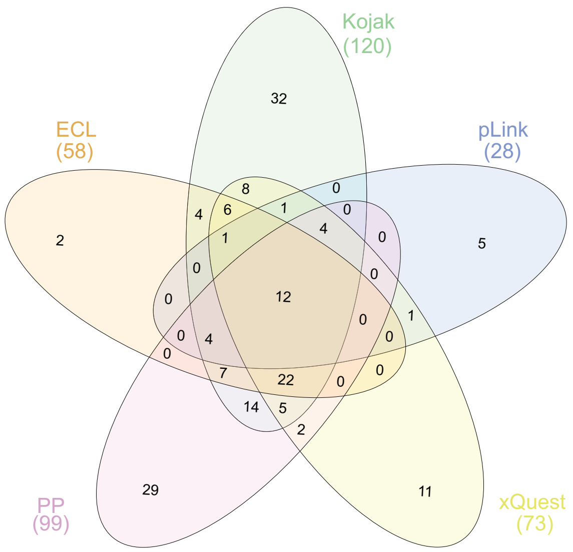

(b) Data set 2

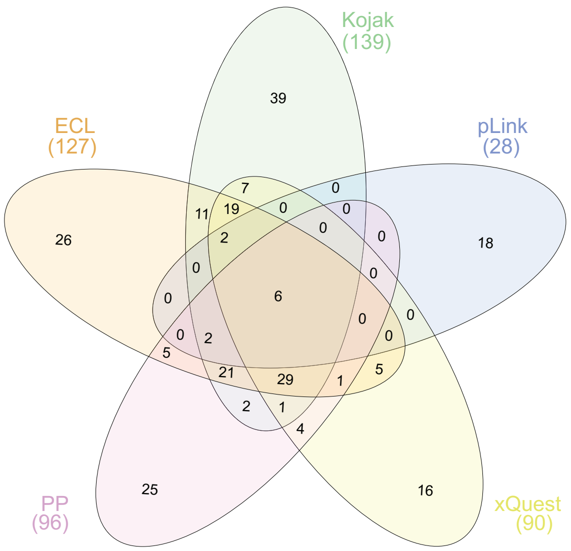

(c) Data set 3

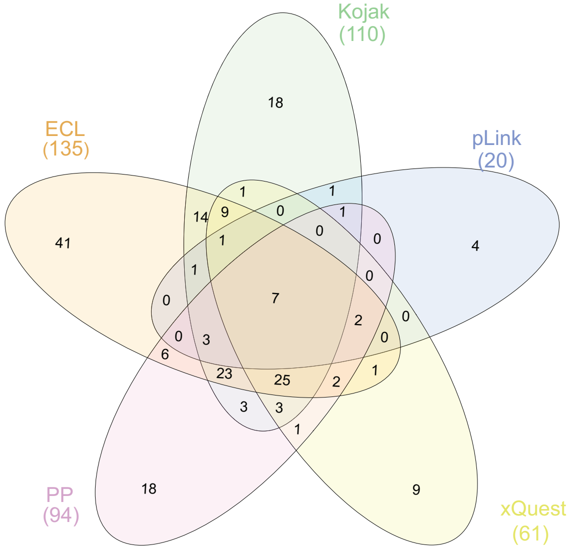

(d) Data set 4

Figure S4: Venn diagrams for 26S proteasome results. PP stands for ProteinProspector.
